# Supplementary material for: ‘We felt like part of a production system’: A qualitative study on women’s experiences of mistreatment during childbirth in Switzerland
Source: PLoS One. 2022 Feb 18;17(2):e0264119. doi: 10.1371/journal.pone.0264119 (PMC8856555; doi:10.1371/journal.pone.0264119)
Supplement: S3 Table — (DOCX) [file pone.0264119.s003.docx]

**S3 Table. Overview of all themes and codes.**

| **Themes** | | **Survey codes^1^** | **Interview codes^1^** |
| --- | --- | --- | --- |
| Bohren et al. themes | | | |
|  | Physical abuse/sexual abuse | Assault | - |
|  | Verbal abuse | Insults, intimidation, causing guilt, threats | Threats |
|  | Failure to meet professional standards of care | Lack of consent, woman justifies lack of consent, pressure, vaginal examinations, cardiotocography (CTG), fundal pressure during delivery, fundal pressure for placenta expulsion, membrane stripping, inadequate pain relief, lack of support | Pressure, lack of informed choice, lack of informed consent, feeling abandoned, neglect, having to wait, painful vaginal examinations, lack of privacy |
|  | Poor rapport between women and providers | Not taken seriously, woman justifies not being taken seriously, lack of information, degradation, unprofessional conduct, lack of collaboration between HCP, rough treatment, language issues, restriction of freedom, lack of respect for wishes/feelings/needs | Lack of interpersonal fit between woman and HCP, unprofessional conduct, degradation, not taken seriously, lack of transparency, conflicting information, lack of information, feeling passive, uncomfortable birthing position, lack of individualised care, lack of respect for needs, discouragement, medication in lieu of support, lack of empathy |
|  | Health system conditions and constraints | Inadequate facility/resources | Hectic atmosphere, facility overstretched, staff stressed, staff out of their depth, shift change, no childbirth debriefing |
| Interview themes and sub-themes | | | |
|  | Informal coercion |  | Threats, pressure, lack of informed choice, lack of informed consent, lack of privacy, not taken seriously, painful vaginal examinations |
|  | Risk factors for mistreatment |  | Inadequate antenatal classes, feeling insecure, helplessness, lack of coping, no longer being able to think, prolonged labour, partner persuades woman, trust issues with HCP |

| Interview themes and sub-themes, continued | | | |
| --- | --- | --- | --- |
|  | Consequences of mistreatment |  |  |
|  | Immediate reactions |  | Assertiveness, opposition, negotiating a compromise, accepting HCP’s opinion, giving in |
|  | Birth experience |  | Negative birth experience, loss of control, feeling at HCP’s mercy, panic |
|  | After-effects |  | Guilt, self-deprecation, self-doubt, thinking about the birth, «what if» thoughts, need for clarification, attitude change, frustration, sadness, regret, thoughts concerning future birth, avoidance |
|  | Coping |  | Helpful childbirth debriefing, processing, extenuation |
|  | Good examples of care |  | One-on-one care, empathetic care, respecting boundaries, informed choice, autonomy, transparent communication, providing time |
|  | What’s needed |  | What’s needed |
| Miscellaneous^2^ | | | |
|  | Childbirth debriefing | Not helpful, not offered, HCP not available, transfer, extra charge, timing, not yet |  |
|  |  |  | Fear of caesarean section, fear of vaginal birth, exceptional situation, negative experience with CTG, straightforward communication, involving partner, social norms, separation from child, preparation |

^1^ The codes were only translated from German to English for the purpose of the present publication.

^2^ Miscellaneous codes were not included in the analyses because they were not relevant for the present research and/or because they were neither positive nor negative evaluations.
