# Supplementary material for: ‘We felt like part of a production system’: A qualitative study on women’s experiences of mistreatment during childbirth in Switzerland
Source: PLoS One. 2022 Feb 18;17(2):e0264119. doi: 10.1371/journal.pone.0264119 (PMC8856555; doi:10.1371/journal.pone.0264119)
Supplement: S1 Appendix — (DOCX) [file pone.0264119.s004.docx]

**S1 Appendix. Interview guide.**

[Table A. English interview guide. 1](#_Toc79504333)

[Table B. German interview guide. 3](#_Toc79504334)

[Table C. French interview guide. 5](#_Toc79504335)

# **Table A. English interview guide.**

| **Key question** | **Checklist and prompts for further questions** | **Directive questions** |
| --- | --- | --- |
| Birth experience | | |
| I would like to begin with your birth experience. Can you tell me about how your birth unfolded from the moment you arrived at the facility? | - Did the birth go more or less as planned or as you had envisioned it?   - Why yes/no? - How did you feel during the birth? - Was there anything that didn’t go so well?   - Was there a specific moment, when the atmosphere changed, you no longer felt comfortable or the situation got out of hand?^1^ | - Nonverbal - Can you tell me more about that? - What happened next? - What about …? |
| Interactions with healthcare professionals | | |
| My next question concerns your interactions with the healthcare professionals during birth (midwives, doctors, possibly students). What did you think of the care provided by the healthcare professionals? | - Did you feel respected? - Did you feel taken seriously? (Regarding your feelings, wishes, needs) - Did you feel supported at all times? - What did you think of the communication and collaboration among the different healthcare professionals? | - Nonverbal - Can you tell me more about that? - What happened next? - What about …? |
| Decision-making | | |
| Next, I would like to know more about the decision-making process during your birth. Please think of a situation where an important decision had to be made. How did that decision come about? | - Did the healthcare professionals include you in the decision-making process? - Was it important to you to have an active say during your birth? - Did the healthcare professionals always inform you of their plans and of every step of their procedure? - Could you always understand what they were doing and why they were doing it? - Did you have enough time to consider the decision? - Did the healthcare professionals always ask for your consent? - Who made the final decision? - How did you weigh the options, what were your thoughts and feelings regarding the decision? - Did you always trust the healthcare professionals? - Did you and the healthcare professionals ever disagree on anything?   - What did you disagree on?^1^   - Did you express your opinion?^1^     - If yes: How did the healthcare professionals react?^1^     - If no: Why not?^1^   - Can you tell me more about the communication with the healthcare professionals?^1^     - What were their arguments?^1^     - Were there different consequences depending on which decision was made?^1^   - Who convinced who? Or did you find a third solution?^1^ | - Nonverbal - Can you tell me more about that? - What happened next? - What about …? |
| Processing the birth | | |
| Lastly, I would like to know how you look back on your birth today. | - Is there anything about your birth that is still on your mind today? - What was most helpful to you in processing your birth experience? - Did you have the opportunity to discuss the birth with any of the healthcare professionals involved after the birth?   - If yes:     - Was it helpful?^1^     - Why yes/no?^1^     - If no: What was missing?^1^     - If no: What would have helped you?^1^   - If no:     - Why not?^1^     - Did they offer you the opportunity?^1^ - Did you see the need? | - Nonverbal - Can you tell me more about that? - What happened next? - What about …? |
| End | | |
| We’ve reached the end of the interview. Is there anything we haven’t talked about that you’d like to add? |  |  |

^1^ Only asked if indicated.

# **Table B. German interview guide.**

| **Leitfrage** | **Checkliste und Stichworte für Nachfragen** | **Aufrechterhaltungs- und Steuerungsfragen** |
| --- | --- | --- |
| Geburtserlebnis | | |
| Als erstes bin ich daran interessiert zu erfahren, wie Sie Ihre Geburt erlebt haben.  Wie war der Verlauf der Geburt ab Eintritt in das Spital/Geburtshaus? | - Verlief die Geburt in etwa so, wie Sie sie sich vorgestellt oder gewünscht haben?   - Gründe warum ja/nein? - Wie haben Sie sich während der Geburt gefühlt? - Gibt es Dinge, die nicht gut gelaufen sind?   - Gab es einen bestimmten Zeitpunkt, wo die Situation «kippte»? Wo Sie sich auf einmal unwohl fühlten / irgendetwas nicht mehr stimmte / die Situation aus dem Ruder lief?^1^ | - Nonverbal - Können Sie dazu noch etwas mehr erzählen? - Und dann? - Wie ging es weiter? - Wie war das so mit …? |
| Interaktion mit Fachpersonen | | |
| Als nächstes interessiert mich die Beziehungsgestaltung mit den Fachpersonen unter der Geburt (also Hebammen, Ärztinnen/Ärzte).  Wie empfanden Sie die Betreuung durch die Fachpersonen? | - Fühlten Sie sich von den Fachpersonen respektiert? - Fühlten Sie sich von den Fachpersonen ernst genommen (bzgl. Ihren Gefühlen, Empfindungen, Wünschen, Bedürfnissen) - Fühlten Sie sich zu jedem Zeitpunkt unterstützt? - Wie empfanden Sie die Kommunikation und die Zusammenarbeit zwischen den verschiedenen Fachpersonen? | - Nonverbal - Können Sie dazu noch etwas mehr erzählen? - Und dann? - Wie ging es weiter? - Wie war das so mit …? |
| Entscheidungsfindung | | |
| Nun würde ich gerne über die Entscheidungsfindung während der Geburt sprechen.  Vergegenwärtigen Sie sich einen Moment während der Geburt, in dem eine wichtige Entscheidung getroffen werden musste.  Wie kam die Entscheidung zustande? | - Haben die Fachpersonen Sie in die Entscheidung einbezogen? - War es Ihnen wichtig, den Verlauf der Geburt mitbestimmen zu können? - Wurden Sie stets über das geplante Vorgehen resp. den nächsten Schritt informiert? - Konnten Sie immer verstehen, was weshalb gemacht wurde? - Hatten Sie genug Bedenkzeit für die Entscheidung? - Wurde immer Ihr Einverständnis eingeholt? - Wer hat die finale Entscheidung getroffen? - Wie haben Sie die Entscheidung abgewogen, was waren Ihre Gedanken und Gefühle dazu? - Haben Sie den Fachpersonen stets vertraut? - Waren Sie jemals bzgl. einer Entscheidung anderer Meinung als die Fachpersonen?   - Worum ging es?^1^   - Haben Sie Ihre Meinung geäussert?^1^     - Wenn ja: Wie haben die Fachpersonen darauf reagiert?^1^     - Wenn nein: Warum nicht?^1^   - Wie lief die Kommunikation mit den Fachpersonen ab?^1^     - Wie haben die Fachpersonen argumentiert?^1^     - Wurden Ihnen mögliche Konsequenzen der Entscheidung aufgezeigt?^1^   - Wer konnte wen überzeugen? Oder wurde eine dritte Lösung gefunden?^1^ | - Nonverbal - Können Sie dazu noch etwas mehr erzählen? - Und dann? - Wie ging es weiter? - Wie war das so mit …? |
| Verarbeitung der Geburt | | |
| Als letztes bitte ich Sie um einen Rückblick: Wie schauen Sie auf Ihre Geburt zurück? | - Gibt es etwas von dieser Geburt, was sie auch heute noch beschäftigt? - Was hat Ihnen am meisten geholfen, die Geburt zu verarbeiten? - Hatten Sie ein Nachgespräch mit beteiligten Fachpersonen?   - Wenn ja:     - War es hilfreich?^1^     - Warum ja / nein?^1^     - Wenn nein: Was hat Ihnen gefehlt?     - Wenn nein: Was hätte Ihnen geholfen?   - Wenn nein:     - Warum nicht?^1^     - Gab es ein Angebot?^1^ - Hatten Sie das Bedürfnis? | - Nonverbal - Können Sie dazu noch etwas mehr erzählen? - Und dann? - Wie ging es weiter? - Wie war das so mit …? |
| Ende | | |
| Wir kommen nun zum Ende des Interviews. Habe ich etwas vergessen, das Sie gern noch ansprechen würden? |  |  |

^1^ Only asked if indicated.

# **Table C. French interview guide.**

| **Question clé** | **Liste et mots-clés pour questions supplémentaires** | **Maintenir et orienter** |
| --- | --- | --- |
| Expérience de l’accouchement | | |
| Tout d’abord, j’aimerais bien savoir comment vous avez vécu votre accouchement. Comment s’est déroulé l’accouchement à partir du moment où vous êtes arrivée à l’hôpital/la maison de naissance ? | - Est-ce que l’accouchement s’est déroulé à peu près comme prévu ou comme vous l’imaginiez ou souhaitiez ?   - Pourquoi oui/non ? - Comment vous sentiez-vous pendant l’accouchement ? - Y a t-il des choses qui ne se sont pas bien passées ?   - Y avait-il un moment où la situation a basculé ? Où vous ne vous sentiez plus à l’aise / où quelque chose selon vous n’était plus juste ?^1^ | - Non verbal - Pourriez-vous m’en raconter plus ? - Et puis ? - Comment cela a continué ? - Et qu’est-ce qui s’est passé par rapport à … ? |
| Contact avec les spécialistes | | |
| Ma prochaine question concerne votre contact avec les spécialistes lors de l’accouchement (sages-femmes, médecins). Comment ressentiez-vous la prise en charge par les spécialistes ? | - Vous sentiez-vous respectée par les spécialistes ? - Vous sentiez-vous prise au sérieux ? (Par rapport à vos sentiments, vœux, besoins) - Vous sentiez-vous soutenue à chaque moment ? - Comment ressentiez-vous la communication et la collaboration entre les différent·e·s spécialistes ? | - Non verbal - Pourriez-vous m’en raconter plus ? - Et puis ? - Comment cela a continué ? - Et qu’est-ce qui s’est passé par rapport à … ? |
| Prise de décision | | |
| Maintenant, j’aimerais bien parler de la prise de décision lors de l’accouchement. Pensez à une situation pendant l’accouchement, où il était nécessaire de prendre une décision importante. Comment la décision s’est-elle prise ? | - Est-ce que les spécialistes vous ont inclue dans la prise de décision ? - Est-ce que ça vous importait d’avoir votre mot à dire au sujet du déroulement de l’accouchement ? - Est-ce qu’on vous a toujours informée du procédé planifié et de chaque étape ? - Est-ce que vous pouviez toujours comprendre ce qui était en train d’être fait et pourquoi il était en train d’être fait ? - Aviez-vous assez de temps pour réfléchir à la décision à prendre ? - Est-ce qu’on a toujours demandé votre consentement ? - Qui a pris la décision finale ? - Comment avez-vous pesé la décision, quel(le)s étaient vos pensées et vos sentiments par rapport à la décision ? - Faisiez-vous toujours confiance aux spécialistes ? - Y avait-il jamais une décision dont vous et les spécialistes n’étaient pas du même avis ?   - De quoi s’agissait-il ?^1^   - Avez-vous exprimé votre avis ?^1^     - Si oui : Comment ont réagi les spécialistes ?^1^     - Si non : Pourquoi ?^1^   - Comment s’est déroulé la communication avec les spécialistes ?^1^     - Quels étaient leurs arguments ?^1^     - Y avait-il des différentes conséquences qui dépendaient de la décision prise ?^1^   - Qui a convaincu qui ? Ou est-ce que vous avez trouvé une autre solution ?^1^ | - Non verbal - Pourriez-vous m’en raconter plus ? - Et puis ? - Comment cela a continué ? - Et qu’est-ce qui s’est passé par rapport à … ? |
| Digérer l’accouchement | | |
| Ma dernière question est : Comment repensez-vous à votre accouchement ? | - Y a t-il quelque chose qui vous travail encore ? - Qu’est-ce que vous a aidé le plus à digérer l’accouchement ? - Avez-vous pu discuter de votre accouchement avec quelqu’un des spécialistes participant·e·s après l’accouchement ?   - Si oui:     - Est-ce que cela vous a aidé ?^1^     - Pourquoi oui / non ?^1^     - Si non: Qu’est-ce que vous a manqué ?     - Si non: Qu’est-ce que vous aurait aidé ?   - Si non:     - Pourquoi ?^1^     - Auriez-vous eu l’opportunité (d’en parler avec un/une des specialistes participant.e.s) ?^1^ - En avez-vous eu besoin ? | - Non verbal - Pourriez-vous m’en raconter plus ? - Et puis ? - Comment cela a continué ? - Et qu’est-ce qui s’est passé par rapport à … ? |
| Fin | | |
| Nous sommes arrivées à la fin de l’interview. Est-ce que vous aimeriez ajouter quelque chose ? |  |  |

^1^ Only asked if indicated.
